# Measuring care of the elderly: psychometric testing and modification of the Time in Care instrument for measurement of care needs in nursing homes

**DOI:** 10.1186/1471-2318-8-22

**Published:** 2008-09-25

**Authors:** Kajsa BE Thorsell, Berit Nordström, Per Nyberg, Bengt V Sivberg

**Affiliations:** 1 Department of Health Sciences, Section of Nursing, Faculty of Medicine, Lund University, Lund, Sweden; 2 Municipality of Hässleholm, Section of Elderly, Hässleholm, Sweden

## Abstract

**Background:**

Aging entails not only a decrease in the ability to be active, but also a trend toward increased dependence to sustain basic life functions. An important aspect for appropriately elucidating the individual's care needs is the ability to measure them both simply and reliably. Since 2006 a new version of the Time in Care needs (TIC-n) instrument (19-item version) has been explored and used in one additional municipality with the same structure as the one described in an earlier study.

**Methods:**

The TIC-n assessment was conducted on a total of 1282 care recipients. Factor analysis (principal component) was applied to explore the construct validity of the TIC-n. Cronbach's alpha was calculated to test reliability and for each of the items remaining in the instrument after factor analysis, an inter-rater comparison was carried out on all recipients in both municipalities. Independently of each other, a weighted Kappa (K_w_) was calculated. Results. The mean of each weighted Kappa (K_w_) for the dimensions in the two municipalities was 0.75 and 0.76, respectively. Factor analysis showed that all 19 items had a factor loading of ≥ 0.40. Three factors (General Care, Medical Care and Cognitive Care) were created.

**Conclusion:**

The TIC-n instrument has now been tested for validity and reliability in two municipalities with satisfactory results. However, TIC-n can not yet be used as a golden standard, but it can be recommended for use of measurement of individual care needs in municipal elderly care.

## Background

Aging entails not only a decrease in the ability to be active, but also a trend toward increased dependence to sustain basic life functions. An important factor for appropriately elucidating an individual's care needs is the ability to measure them both simply and reliably. Researchers have compared the situation in Europe, Asia, and the US to describe the care needs of the individual care recipient and the resources required to meet them [1-5]. Several researchers [6-10] have validated various measurement systems for care needs in the elderly and have also illuminated the importance of developing consistent measuring methods for routine use by healthcare personel. On the international level, different methods have been developed to measure care needs, but these are usually tailored to the local healthcare systems and are related to disease diagnoses. However, they omit many essential areas such as the psychosocial field. The Nordic countries are working hard to develop various measurement instruments to elucidate the needs of care recipients. Researchers are investigating how these instruments can best be used in daily practice, within both regional medical care and municipal primary care. Katz-ADL, which has been further developed in Sweden, intends to measure the degree of independence of activities in everyday life. It consists of six variables with two or three levels. The instrument has the character of steps, where the increase of the care need corresponds with a specific sequence. To reflect the care need within the municipal healthcare system the Katz-instrument was expanded to comprise one section which highlights a number of daily activities such as laundry, cleaning and cooking. Through this expansion, the ADL-scale came into practice within healthcare assessment in Sweden. An additional instrument is The Residential Assessment Instrument (RAI), originally developed in the US and intended for a hospital environment. Care assessment according to RAI is an extensive process encompassing several professions as well as several hundreds of variables. RAI is, in Sweden, mainly utilized by researchers due to its massive time effort. The rehabilitation field is mainly using Functional Independence Measure (FIM), The Berger Scale and Mini Mental Test (MMT) to measure cognitive impairment [11]. In residential care the Rush Medicus, Beakta and Zebra instruments are applied. Recently different reports concerning care assessment have included The EQ5D and The Berthel Scale [11]. These instruments solely measure perceived quality of life. The instruments mentioned above suffer from one inadequacy: they do not measure the psychosocial needs in combination with other needs. This is the main reason why a new instrument has been developed.

A patient dependency classification system is a strategy for categorizing patients according to their nursing care requirements. The purpose of a classification system is to assess patients, categorize them, and allocate them to groups with similar nursing needs. The patients in each group are then given a numerical score to indicate the amount of nursing care they need [12].

In an article from 2006 [13] different models to measure nursing care requirements are described. By counting the number of dependencies or by weighting certain activities of daily living to contribute to an individual's cumulative score, researchers create a score that is intended to reflect an individual's overall functional ability and thus, the person's care needs [14,15]. Howell-White et al [13] describe three general types of models: rules, count, and weighted. Rule models use guidelines that define each tier and the requirements for inclusion.

Individuals are assigned to the category that best describes their condition, limitations, or care needs. Count models assign a score to the need for assistance across the various indicators.

The scores are then totalled and used to tier individuals. Weighted models, like count models, compute total scores across a set of indicators, but each factor is adjusted or weighted by relative importance. Weighting expands the potential range of scores, thereby creating more flexibility within the tiers. Weights can be derived empirically or by expert assessment [16,17].

### The Time in Care instrument

A project [18] that has been underway for about 10 years is developing a measurement instrument to assess the care recipient's care needs within municipal elderly care. The name of the instrument is Time In Care (TIC) and it consists of two parts: Time In Care for need (TIC-n), which measures the care needs of the individual, and Time In Care for time (TIC-t), which measures the number of daytime hours that the nursing staff devote to meeting the individual's care needs. An initial study originally consisting of 25 items in the TIC-n was able to reduce the number of items with the help of factor analysis [18]. Development of this instrument continues, since professional experience demanded five new items to be added and two items needed to be excluded. In total 19 items of TIC-n were used in this study. The purpose was also to further validate TIC-n using new material from two Swedish municipalities, as well as to investigate the reliability between measurements. The version of TIC-n used for data collection in this study consists of these 19 items divided into three need dimensions: *General Care *(9 items), *Medical Care *(5 items) and *Cognitive Care *(5 items), (Table 1).

**Table 1:** The TIC-n instrument

Unit........................	Ward.......................			Date........	
Code caretaker................	Assessment made by....................				
A. General Core					

Items/point	0 point	1 point	2 points	3 points	4 points

Nutrition					
Washing upper body					
Washing lower body					
Toilet visit					
Dressing/undressing					
Shower/bath					
Mobilization					
Observation/supervision/alarm					
Social activities					
Total					

B. Medical care					

Items/points	0 point	1 point	2 points	3 points	4 points
Wound treatment					
Catheter/stoma					
Administration					
Injection					
Rehabilitation					
Total					

C. Cognitive Care					

Items/points	0 point	1 point	2 points	3 points	4 points
Orientation/sense of locality					
Verbal communication					
Confusion					
Anxiety					
Temper					
Total					

TIC-n is used by care staff (nurses, nursing assistants) in order to assess the care need as a part of the individual care plan, and constitutes the foundation for how the care need can be met with the resources required for a satisfactory care. The instrument is also used by management as well as nursing staff, and assessment of the individual care need can be carried out when the care is to be evaluated or revised. Assessment is performed by the assisting nurse and nurse through a clinical observation aided by the assessment form (Table 1) as well as the TIC-manual. In order to make a functional assessment, the assessors need to be perfectly familiar with the instrument and in possession of adequate knowledge of the individual caretaker. Training for new users is given by certified staff that is familiar with both constitution and field of application of the instrument.

## Methods

The TIC-n instrument, which is a count model, includes a number of needs with established items structured from 0 – 4 points. These describe the degree of need present based on an established manual. Changes were made to the 16 items that resulted from the factor analysis in the first version [18]. In the version of TIC-n used in this study 14 items from the first version were used and five new items were added (Supervision/alarm, Administration of drugs, Rehabilitation, Anxiety, Temper and Confusion). The reasons of the extension were that the 16 items from the first version did not mirror all the needs of the individual caretaker. Considerations were made to add five new items and a new test version of TIC-n with 19 items was established. A consequence of that change was that the TIC-n manual for user instruction needed to be revised. The manual has continuously been revised to make it more consistent and easier to understand for users. Staff has been instructed in the manual by training and supervision. The total number of points (range 0 – 76 points) is divided into five levels of care, which comprise intervals of the current care need inside which the care recipient is found. This categorization of care needs into five levels of care follows the WHO classification system for documenting various disabilities and health (Table 2).

**Table 2:** Points given in care levels

**Level**	**Points**	**Term**
1	0–11	Little or no caring need (help with: bath, shower, cleaning, shopping etc)
2	12–23	Moderate caring need (help with: shower/bath, attention once a day in personal care, administration of drugs, cleaning)
3	24–33	Increased caring need (help several times a day with personal care. Can't go to the toilette by themselves, help with dressing, shower/bath and social services)
4	34–43	Very much increased caring need (can't do anything in the daily care, problem with cognitive dysfunction, medical care, can't live by themselves)
5	44 +	Totally increased caring need (totally help in all four factors, palliative care)

Note. The scale scores are given in range 0–4 points in the instrument.

**Figure 1:**
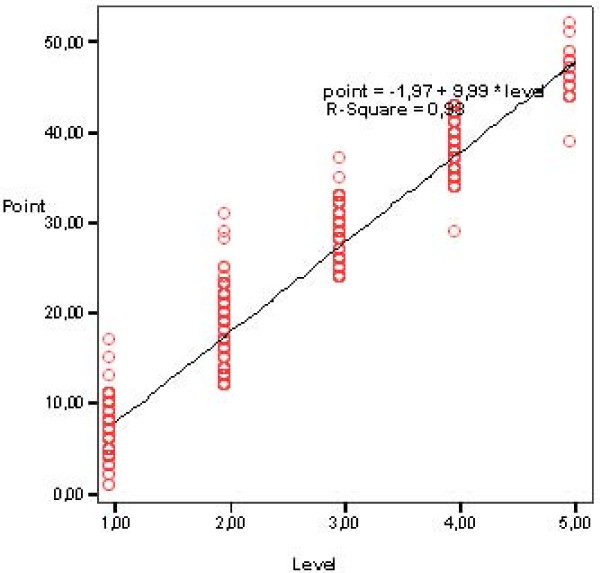
Distribution of care recipients (five levels) of care needs in the 16 items version of TIC-n.

The cut points for the care levels are set according to the distribution of care recipients presented in a scatter plot diagram. There is a group of outliers in all levels, but the majority of care recipients in all five levels are distributed close to the cut points (Fig. 1).

The TIC-n instrument (19-item version) has been used in one additional municipality with the same structure as the one described. A total of 31 nursing homes were assessed during 2007 using TIC-n in accordance with the established manual. The need assessment was conducted on a total of 1282 care recipients with an average age of 86 years (range 40 – 101 years), 74 percent of whom were women. Average length of stay was 1.5 years (range 5 – 36 months).

All statistical analyses were performed using SPSS 11.5 (SPSS Inc, Chicago, USA). Validity was explored by factor analysis of the TIC-n construct (principal component analysis with varimax rotation). Items to be included in the factor structure had to meet the criterion of a factor loading of ≥ 0.4 [19]. Logistic regression analysis was performed in order to establish the scale levels of care needs in TIC-n in order to explore the discriminating capacity of the scale.

Reliability was investigated by Cronbach's alpha and explained variance was calculated. For each of the items remaining in the instrument after factor analysis, an inter-rater comparison was carried out on all (n = 1282) recipients in both municipalities. Each care recipient was rated twice by a nurse assistant and an independent observer, who was a registered nurse not working at the ward. At the same point in time, but independently of each other, a weighted Kappa (*K*_w_) was calculated according to Altman [19]. In order to compare the results of the weighted Kappa between the two municipalities, these will be reported in part separately for the two municipalities and also in total for the entire material.

## Ethical considerations

The care recipients and their relatives as well as the staff were verbally informed about the study and efforts were made to prevent any worry over the observations, after which they all gave their informed consent for participation. The Regional Ethics Committee, Faculty of Medicine at Lund University (LU-321-03), deemed that no further formal inquiry was needed, since the study could be viewed as routine quality assurance. The study was fully compliant with the Declaration of Helsinki, which states that all information must be conveyed to the care recipients.

## Results

### Inter-reliability and stability

The mean of each weighted Kappa (*K*_w_) for the dimensions in the two municipalities was 0.75 and 0.76, respectively, showing close overall agreement between the two rates [20]. The *K*_w _values ranged from 0.50 to 0.97, the lowest value (0.50) pertaining to the *Medical Care *item regarding need for Rehabilitation and the highest value (0.97) pertaining to the item regarding Catheter and Stoma (Table 3).

**Table 3:** Inter-rater test of Tic-n with K-value and data from two municipalities, and merged data

	Municipality	
	1	2	Merged data
	n = 453	n = 829	n = 1282
A:1 Nutrition	0,83	0,79	0,81
A:2 Washing upper boy	0,63	0,82	0,72
A:3 Washing lower body	0,92	0,86	0,89
A:4 Toilet visits	0,88	0,87	0,87
A:5 Dressing/undressing	0,84	0,85	0,85
A:6 Shower/Bath	0,72	0,80	0,76
A:7 Mobilization	0,85	0,79	0,82
A:8 Supervision/alarm	0,65	0,69	0,67
A.9 Social activities	0,74	0,70	0,72
B:1 Wound treatment	0,68	0,65	0,66
B:2 Catheter/stoma	0,97	0,72	0,84
B:3 Administration of drugs	0,74	0,70	0,72
B.4 Injection	0,87	0,72	0,79
B:5 Rehabilitation	0,50	0,69	0,59
C.1 Orientation/sense of locality	0,52	0,76	0,64
C.2 Verbal communication	0,82	0,70	0,76
C:3 Confusion	0,75	0,72	0,73
C:4 Anxiety	0,61	0,66	0,63
C:5 Temper	0,90	0,69	0,79
			
Total Kappa value	0,76	0,75	0,75

### Factor analysis and construct validity

Factor analysis showed that all 19 items had a factor loading of 0.40 (Table 4).

**Table 4:** Factor analysis matrix of TIC-n. data from 1282 care recipients in two municipalities

		Factors		
	General Care	Cognitive Care	Medical Care	Communalities
	Load	Load	Load	
A:7 Mobilization	0,836			0,733
A:5 Dressing/undressing	0,812			0,822
A:3 Washing lower body	0,783			0,807
A:4 Toilet visits	0,773			0,795
B:1 Wound treatment	0,709			0,529
A:6 Shower/bath	0,694			0,651
A:2 Washing upper body	0,693			0,785
B.5 Rehabilitation	0,690			0,653
A.1 Nutrition	0,633			0,677
B:3 Administration of drugs	0,567			0,597
B.4 Catheter/stoma	0,448			0,230
C:4 Anxiety		0,845		0,814
C.1 Orientation/sense of locality		0,784		0,768
C:5 Temper		0,689		0,429
C:2 Verbal communication		0,666		0,634
C:3 Confusion		0,651		0,814
A:8 Supervision/alarm		0,615		0,705
A:9 Social activities		0,551		0,606
B:4 Injection			0,98	0,967
				
% Variance explained	53,99	7,57	5,20	66,76
				
Cronbach's alpha	0,941	0,891		0,949

Note. Factor analysis: principal component, varimax rotation.

**Figure 2:**
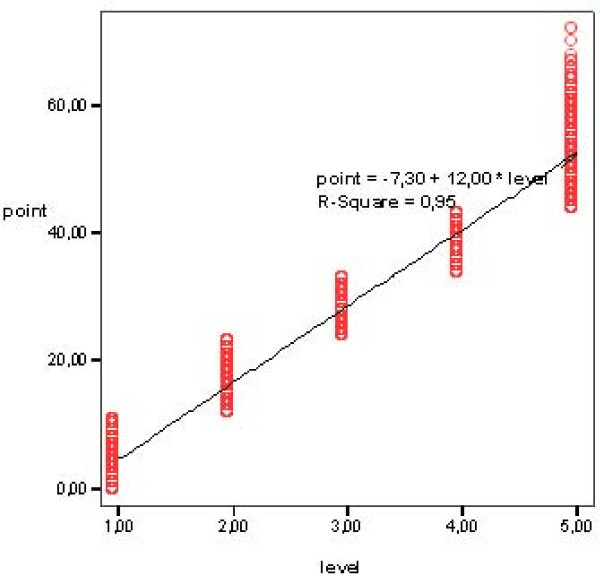
Distribution of care recipients (five levels) of care needs in the 19 items version of TIC-n.

The results show that 11 items were loaded into *General Care *(m = 1.6; range 0.22 – 2.53) and seven items were loaded into *Cognitive Care *(m = 1.4; range 0.58 – 2.33). Under *Medical Care*, Injection comprised a separate item with a factor loading of 0.98. The mean score total for care needs in the sheltered living homes was 27.6 (sd 18.82), while means for the separate homes ranged from 1 to 48.3 points. A total of 26% of the care recipients were classified in Level 1 (m = 5 points; range 0 – 11 points), 20% Level 2 (m = 17 points; range 12–23 points), 15% Level 3 (m = 28 points; range 24 – 33 points), 15% Level 4 (m = 38 points; range 34 – 43 points) and 24% in Level 5 (m = 53 points; range 44–76 points) (Figure 2).

## Discussion

The development of the TIC-n instrument has taken a step forward through this study. To give a reliable picture of daily care activities, two items were reduced from the 16-item version, and five items were added which resulted in total 19 items. Consequently, 14 items from the first version plus 5 new items were used for input in the second factor analysis. Also, this second factor analysis was performed on a much bigger sample. There was a great difference in sample size between the first and second factor analyses, 505 and 1282, respectively. This fact may explain why four items (Wound-treatment, Rehabilitation, Administration of drugs and Catheter/stoma) changed factor dependence (higher load) from *Medical Care *to *General Care*. These four items which changed from *Medical Care *to *General Care *were not distinguished as medical in comparison with Injection which remained in *Medical Care *in the second factor analysis. Also two items (Supervision/alarm and Social activities) changed factor dependence from *General Care *to *Cognitive Care *in the second factor analysis due to their strong connection to behavioural sciences. However, in the future there is a need to further explore the TIC-n instrument with a confirmatory factor analysis in order to establish the stability of the factor structure.

The sampling process of TIC-n data was further improved by a simplified and revised manual to support the reliability, which meant that the revised version consisting of 19 items became more clearly described as well as more clearly defined from one another. The present study has further explored and strengthened the reliability of TIC- n. For each item, an inter-rater comparison was carried out for all care recipients (n = 1282). Each care recipient was rated twice, once by a nurse assistant and once by an independent registered nurse observer. This procedure to use a registered nurse to supervise the assessment of the nursing assistant was taken into account to assure assessment competence of the nursing assistant. The rating process was carried out simultaneously, but independently, in order to avoid biases. For reasons of methodology, weighted Kappa (*K*_w_) was used to assess the agreement between the inter-raters, showing a result of good to almost very good agreement [21]. The procedures performed to test reliability are sufficient to establish reliability for the TIC-n.

In one municipality (n 453) two items, Rehabilitation in *Medical Care *and Orientation in *Cognitive Care *both displayed a low level of agreement (0.50 respectively 0.52), which can be attributed to the difficulty of interpreting these items in a uniform manner. Rehabilitation can be a wide and difficult field to interpret. Rehabilitation does not consist of a separate activity for nursing staff but is included in daily care. In the factor analysis Rehabilitation was therefore transferred to *General Care*. Difficulties in discerning whether the ability of orientation/sense of location is impaired due to cognitive aspects or if it is within the range of normality, also affect the results. It is probable that the staff had difficulties in assessing the caretakers' orientation skills. Improved education/training concerning orientation/sense of location may enhance results in the future.

It is important to conduct an inter-rater comparison of assessments using weighted Kappa analysis at least once annually in order to ensure sufficient reliability. One important basic requirement that must be fulfilled to ensure reliability is that all users must accept the procedures of TIC-n assessment. It must be possible to monitor the assessment process and compliance with the manual. The system does not permit each user or group of users to add or change items. This precludes the ability to compare the information between various wards. It is therefore important that thorough instruction be provided both during implementation and at regular follow-up intervals.

The internal consistency of the TIC-n instrument was determined by means of Cronbach' s alpha. An internal consistency greater than the required value of 0.90 means that the scale may be applied on an individual level [20]. The alpha coefficients (range 0.89 – 0.94) showed that TIC-n has high homogeneity, supporting the internal consistency reliability of the scale.

A factor analysis was carried out to explore whether all items remained in the factor structure after the addition of the five new items to the TIC-n. It showed that eleven items were captured by *General Care *and seven by *Cognitive Care*. This was more than in the first version of TIC-n. Only one item (Injection) was captured in *Medical Care*, which can be explained by the fact that injection can be considered purely medical. Some care recipients have diabetes that requires daily injections of insulin where it is essential that nursing staff have the knowledge to deal with any swings in blood sugar levels that may occur.

## Conclusion

The use of TIC-n allows assessment of individual care needs to be carried out for each care recipient, which is a requirement for individual care planning. The results meet the demands from the Swedish government that requires individual statistics to be reported in order to compare both costs and satisfaction of needs. The instrument is simple to use, accurately reflects the care situation, and is easy to understand. Nursing staff have continuously participated in the TIC-n development process by regularly using of the instrument after revisions were made. The manual currently in use has been tested repeatedly during the data sampling process in the two studies as well as in the pilot study. Because an additional municipality participated in the TIC-n assessment the material was large enough to ensure that the instrument now can be recommended for use in municipal elderly care due to its low time consuming property, accounting to individual care needs and functional allocation of care resources.

## Limitations of the study

A limitation of the study has been the fact that despite further development of the instrument it does not so far allow a "golden standard" of care need assessments. Additional studies need to be carried out in more municipalities in order to compile a sufficiently large material to develop this "golden standard".

## Competing interests

The authors declare that they have no competing interests.

## Authors' contributions

KT was responsible for and involved in all parts of the work with the manuscript. KT was alone responsible for the data collection and the contacts with the nursing staff in the two municipalities. She is a PhD student at Lund University. Together with BN, BVS, and PN she has performed the statistical analysis, the instrument development and the interpretation of results. PN has particularly been involved in the statistical supervision. BN has participated in the elaboration and writing of the manuscript as an assistant supervisor. As the main supervisor BVS was responsible for the design of the study and has participated in all parts of the study and has participated in all parts of the work with the manuscript. All authors critically reviewed and approved the final manuscript.

## Pre-publication history

The pre-publication history for this paper can be accessed here:



## References

[B1] Jamieson A, Illsley R (1990). Contrasting European Policies for Care of Older People. Avebury, Aldershot.

[B2] Wilderom CP, Nijkamp P, Vollering A (1990). Services for elderly in Europe: Across- National Comparative Analysis.

[B3] Rostgaard A, Fridberg T (1998). Caring for Children and Older People. A comparision of European policies and practices.

[B4] Meijer A, van Campen C, Kerkstra A (2000). A comparative study of the financing, provision and quality of care in nursing homes. The approach of four European countries: Belgium, Denmark, Germany and the Netherlands. Journal of Advanced Nursing.

[B5] I-Chuan L, Yin T (2005). Care needs or residents in care-based long- term care facilities in Taiwan. Journal of Clinical Nursing.

[B6] Ebener MK (1985). Reliability and validity basics for evaluation classification systems. Nursing Economics.

[B7] Bennet JA (1990). Activities of daily living: Old fashioned or still useful?. Journal of Gerontological Nursing.

[B8] Kelleher C (1992). Validated indexes: key to nursing acuity standardization. Nursing Economics.

[B9] Kane RL, Kane RA (2000). Validated index: key to nursing acuity standards. Annual Review of Public Health.

[B10] Fagerstrom L (2000). Validation of a new method for patient classification, the Oulu Patient Classification. Journal of Advanced Nursing.

[B11] Larsson T, Goverment S (2008). SOU 2008:51. Värdigt liv i äldreomsorgen. Fritzes kundtjänst.

[B12] Adomat R, Hewison A (2004). Assesing patient category/dependence systems for determining the nurse/patient ratio in ICU and HDU: a review of approaches. Journal of Nursing Management.

[B13] Howell-White S (2006). Creating needs based tiered models for assisted living reimbursement. Gerontologist.

[B14] Travis SS, J MW (1990). Simple counts of the number of basic ADL dependencies for long-term care research and practice. Health Services Research.

[B15] Jette AM (1994). How measurement techniques influence estimates of disability in older populations. Soc Sci Med.

[B16] Finch M, Kane R, Philip I (1995). Developing a new metric for ADL. Journal of the American Geriatrics Society.

[B17] Spector WD (1987). The hierarchical relationship between activities of daily living and instrumental activities of daily living. J Chronic Dis.

[B18] Thorsell KB, Nordström B, Nyberg P, Sivberg BV (2006). Can care of elderly be measured? A method for estimating the individual care of recipients in community health care. BMC-Geriatric.

[B19] Altman D (1991). Practical Statistics for Medical Research.

[B20] Polit DF, Beck CT, Wilkins LW (2006). Essentials of Nursing Research Methods, Appraisal, and Utilization.

